# Sharing and connecting with others – patient experiences of radically open dialectical behavior therapy for anorexia nervosa and overcontrol: a qualitative study

**DOI:** 10.1186/s40337-021-00382-z

**Published:** 2021-03-04

**Authors:** Martina Isaksson, Ata Ghaderi, Martina Wolf-Arehult, Caisa Öster, Mia Ramklint

**Affiliations:** 1 Department of Neuroscience, Psychiatry, Uppsala University, Entrance 10, Floor 3B, SE-751 85 Uppsala, Sweden; 2 Division of Psychology, Department of Clinical Neuroscience, Karolinska Institutet, SE-171 77 Stockholm, Sweden; 3 Stockholm Centre for Eating Disorders, Stockholm Health Care Services, Stockholm County Council, SE-171 77 Stockholm, Sweden; 4 Psychiatry Northwest, Region Stockholm, Clinical Management, PO Box 98, SE-191 22 Sollentuna, Sweden

**Keywords:** Eating disorder, Anorexia, Qualitative analysis, Thematic analysis, Radically open dialectical behavior therapy, RO DBT

## Abstract

**Background:**

Recovery rates after psychological treatments for anorexia nervosa are low to moderate, and in adults, no treatment outperforms any other. The aim of this study was to evaluate patient experiences of Radically open dialectical behavior therapy (RO DBT), a treatment developed for disorders related to maladaptive overcontrol.

**Methods:**

Eleven female patients with anorexia nervosa were interviewed after either treatment completion (eight patients) or drop-out (three patients) from RO DBT. Interviews were transcribed and analyzed with inductive thematic analysis.

**Results:**

The analysis yielded five main themes: 1) a comprehensive treatment, 2) the benefits of sharing and connecting with others, 3) growing trust, 4) moving toward valued goals – but some remain, and 5) doing well in treatment.

**Conclusion:**

Patients appreciated what they described as a comprehensive treatment and holistic view of their problems, which helped them reduce both maladaptive overcontrol and eating disorder symptoms. Gradually sharing personal experiences both in- and outside therapy was described as essential and led to enhanced social connectedness.

**Trial registration:**

The intervention study (Isaksson M, et al. J Behav Ther Exp Psychiatry. 71, 2021) that preceded this interview study was performed by the first, second, third, and fifth author, preregistered in the ISRCTN registry (no: ISRCTN47156042).

**Supplementary Information:**

The online version contains supplementary material available at 10.1186/s40337-021-00382-z.

## Plain English summary

Anorexia nervosa is a severe psychiatric disorder and recovery rates after psychological treatments are low to moderate. Both patients and clinicians have raised criticism of the treatments that are most frequently offered, suggesting that there is too much focus on food and weight, with little emphasis on psychological and social needs and what may have caused the disorder. Excessive overcontrol, characterized by e.g., rigidity, inhibition of showing feelings, and social distancing, has been suggested as an important mechanism for developing the disorder. In the present study, we evaluated how patients that had participated in Radically open dialectical behavior therapy (RO DBT), a psychological treatment developed for disorders related to excessive overcontrol, experienced the treatment. Eleven patients with anorexia were interviewed after they either had completed (eight patients) or dropped out (three patients) from the treatment. Their experiences were recorded and the recordings transcribed to written text. After, the text was analyzed and the content was categorized in overarching themes. Patients appreciated what they described as a comprehensive treatment. They described that the treatment had a holistic view of their problems, beyond mere focus on food and weight, which helped them reduce both maladaptive overcontrol and eating disorder symptoms. Patients also described that they wanted to do well in treatment, and that their trust in the treatment and their therapists improved as therapy progressed. Finally, gradually sharing personal experiences both in- and outside therapy was described as important for recovery.

## Introduction

There is no superior psychological treatment for adults with anorexia nervosa (AN) [1]. Recovery rates are generally low to moderate, varying between around 10 and 40%, and attrition and relapse rates are high [2]. Clinical guidelines recommend a combination of psychotherapeutic treatment and re-nourishment, and that the majority of patients with AN should be treated in outpatient care [1, 3]. However, severe AN or lack of improvement indicate a need for meal support in a day program or inpatient care. Among other things, it has been recommended that new interventions be developed to improve treatment outcome [1, 4].

When evaluating new treatment approaches, experimental designs are essential [5]. However, information on the patients’ own experiences may supplement quantitative data, which is of particular importance in early phases of complex intervention evaluation [6]. Qualitative research may promote a better understanding of treatment effect and fidelity and aid identification of potential mechanisms of change [6]. However, little is known about patient experiences of outpatient treatments for AN. Patients often experience that AN treatments in general (both in- and outpatient) are too rigid, with an excessive focus on food and weight [7] and too little emphasis on psychological and social needs and what may have caused the disorder [8, 9]. Some researchers have raised similar criticisms, suggesting that the treatments most frequently offered focus too much on symptom reduction, while failing to address the core psychopathology of AN [10–12].

Overcontrol has been suggested as a potentially important mechanism for development and maintenance of AN [13, 14]. While self-control, or overcontrol, is defined as the ability to inhibit emotional urges, impulses, and behaviors to pursue long-term goals [15, 16], *excessive* overcontrol is when self-control, emotion inhibition, and rigidity reach a degree that severely affects the individual’s well-being [16]. Radically open dialectical behavior therapy (RO DBT) is a treatment developed for patients with psychiatric disorders linked to excessive overcontrol difficulties. In RO DBT, emotional loneliness is regarded as the core problem, resulting from low openness (e.g., rigidity and dismissing feedback) and deficits in social signaling (e.g., smiling when distressed or inhibited sharing of personal experiences). Thus, both openness and social signaling are considered to be potentially important mechanisms of change to enhance social connectedness and increase well-being [14, 16]. The capacity to openly adapt to situational demands and to express emotions is practiced and reinforced during treatment. Also, an open, self-disclosing, genuine, and relaxed therapeutic stance is used to model adaptive social signaling and increase patient engagement (Lynch [17], page 406).

There have been studies evaluating treatment effect of RO DBT for AN [18–20]. However, no evaluation assessing both treatment effect and patient experiences has been performed. Studies evaluating patient experiences of outpatient AN treatments are few, in particular for treatment approaches with a broader focus than mainly ED symptoms. The aim of the present study was to evaluate patient experiences of RO DBT for AN and overcontrol in a clinical outpatient setting. Details on quantitative results are reported in an experimental, multiple baseline single-case experimental design study that can be found elsewhere [21], preregistered in the ISRCTN registry (no: ISRCTN47156042, URL: https://www.isrctn.com/ISRCTN47156042).

## Methods

### Research design

Patient experiences were evaluated using an inductive qualitative thematic analysis as described by Braun and Clarke [22]. Analyses were performed using the following steps: familiarizing with the text, coding, generating themes, reviewing themes, defining and naming themes, and writing and exemplifying themes. The method was chosen because it is suitable for evaluating patient experiences, and because it is flexible and appropriate for managing rich data material.

### Participants, setting, and procedure

All thirteen patients who had participated in the experimental evaluation of RO DBT for AN at the eating disorder (ED) clinic at Uppsala University Hospital were eligible for participation. Because the study sought to evaluate the effects of outpatient treatment, only patients with a BMI ≥ 16 kg/m^2^ were included, as patients with severe AN are generally treated with inpatient care or with extensive meal support in a day program. In addition, because RO DBT is only relevant for patients with overcontrol tendencies, each patient’s personality style was identified based on the prototype rating by Lynch ([17], p. 81–82). Excluded were patients 1) in need of intense care for ED or treatment for another disorder, 2) participating in psychological treatment within three months before study start, or 3) with cognitive difficulties or difficulties with the Swedish language, based on if a patient could not independently fill out questionnaires or answer questions during interviews.

A research assistant not involved in the treatment contacted the patients via phone to provide information about the qualitative interview. Each patient who was willing to participate was booked for a face-to-face interview within a month from treatment termination. Eleven patients accepted – all eight completers and three out of five non-completers from the single-case study of treatment effect. Written informed consent was obtained from all 11 participants.

Participants were diagnosed with mild to moderate AN (ten patients) or atypical AN (one patient). Full criteria for AN and atypical AN diagnoses were met at the start of treatment. Mean age was 25 years, standard deviation (*SD*) = 6.02, range 18–41. Mean ED onset was at 15 years of age (*SD* = 2.64), range 10–20. The mean duration of the ED was 9 years (*SD* = 6.42), range 1–27. The number of comorbid diagnoses was between one and three. All eight completers were in remission after treatment, with BMI within a healthy range and ED psychopathology within one SD of the community mean. The non-completers were either in partial remission or not improved. Ethical approval was obtained by the Regional Ethics Committee in Uppsala (Ref. no. 2014/252).

### The treatment

RO DBT is a transdiagnostic treatment developed for people with excessive overcontrol, identified in a spectrum of disorders such as AN, chronic depression, and cluster A and C personality disorders [14, 17]. Long-term goals in RO DBT are to form warm and intimate relationships and to reduce emotional loneliness. RO DBT differs from other treatments for AN. For example, rather than mainly focusing on weight restoration and ED symptoms – openness, flexibility, and social signaling are emphasized as key mechanisms of the treatment. A comprehensive description of the RO DBT treatment is provided in the textbook [17] and skills training manual [23] by professor Lynch. Material essential for providing RO DBT was translated to Swedish and used in the present study [24, 25].

The RO DBT intervention at the ED clinic in Uppsala was performed in outpatient care. It consisted of two phases: 1) an initial engagement phase of 6 weeks, orientating the patient to the RO DBT treatment and taking the first steps toward healthy eating and weight, and 2) 34 weeks of both individual therapy and skills class, focusing on enhancing openness, flexibility and social connectedness.

### The interviews

A semi-structured interview guide was developed to explore the patients’ overall experiences of the treatment, and began with an open question asking participants to describe their experiences. Subsequent questions covered the experiences of the treatment’s contents and structure, the therapeutic relationship, potential differences from previous treatments, potential areas for improvement of the treatment, and the participant’s changes or lack of changes during treatment. Probing questions were used throughout each interview to get the participant to develop their answers or give examples. Interview time ranged between 25 and 85 min. A research assistant who had training and experience in interview technique performed the interviews. The interviewer had no involvement in the preceding treatment, and had no knowledge of the outcome for any of the patients. Prior to the interviews, subjects were informed that interviews would be anonymized, with identifiers removed before analyses. Interviews were audio-recorded and transcribed verbatim by another research assistant not otherwise involved in this project. Identifiers such as patient and therapist names were removed.

### Data analysis

To ensure methodological quality, transcripts were reviewed separately by two independent reviewers, the first author (one of the RO DBT therapists) and the fourth author (with extensive experience in qualitative analyses and no involvement in the ED clinic). First, interviews were read and reread to give the reviewers familiarity with the contents. Second, text relevant for the research question and aim was coded with appropriate labels. Third, themes and subthemes were generated by identifying patterns among the labels. To make sure the themes represented the data, interviews were reread several times. Fourth, the first and fourth author compared their findings, discussed and resolved any differences, and came up with preliminary names for the themes. Fifth, the process of reviewing the themes, going back to the interviews, recoding under the new themes, and discussing was performed a second time. Lastly, themes with codes were presented to the other authors and disagreements were discussed until agreement was achieved.

Because we only had a limited number of potential participants (i.e., 13), analysis of data saturations was not used in the recruitment procedure. However, no new categories emerged when going through the last interviews.

## Results

Five main themes were identified: 1) a comprehensive treatment, 2) the benefits of sharing and connecting with others, 3) growing trust, 4) moving toward valued goals – but some remain, and 5) doing well in treatment. Between two and four subthemes were identified within each main theme. Themes and subthemes are described below and presented in a summary in the Additional file 1.

### A comprehensive treatment

#### A flexible and complex approach

Patients expressed appreciation for the fact that the treatment was intense and comprehensive with a holistic view on their problems, both in response to the initial open question and when they contrasted it to previous treatments. The treatment was also experienced as flexible, with interventions adapted to individual needs.

*I felt that this treatment is more thorough than previous treatments … you see the whole person and nothing fell through the cracks … you’re not only focusing too narrowly on the eating disorder. (Participant 9)*

Patients also said they liked that RO DBT included both individual therapy and skills training. Some highlighted that it was helpful with text message contact between sessions, others appreciated that contact through text messaging was available, even if they did not use it. Individual therapy was described as essential for adapting the therapy to fit individual needs. Descriptions mentioned that the treatment dealt with the ED from multiple perspectives, that it was adequately long, and that the balance between working with overcontrol in general, such as social signaling, relationships and emotions, and working with ED symptoms, was helpful.

*There has been a lot of focus on feelings and relationships, which is very different from previous treatments. I’ve been to day programs several times, focusing only on eating, but that has not been therapy … and once I went to regular therapy with no focus on the food - which made me sink like a stone. This therapy has been a good balance. I was allowed to talk about food if I felt “I have to talk about this and get a structure!” … It’s been a very good combination. (Participant 4)*

Even though most participants highlighted the flexibility of the treatment as a strength, one description included a wish for even more flexibility. It was also mentioned that severe ED symptoms, such as starvation, were sometimes an obstacle to working with overcontrol and participating in the skills class, as they made it hard to grasp the essence and the sometimes demanding material and training. Both the lack of flexibility in receiving more help if needed, and the preoccupation with food and weight, was experienced as a hinder for completing the treatment. Some experienced the contents and the materials in skills class as extensive or complicated, while others felt they were overly simplified.

*It was hard to focus on other things because I was so caught up in the eating disorder and everything else became less interesting, I was kind of hijacked by the eating disorder … I think it would be easier if you are more stable. (Participant 1)*

#### Following, or not following, the treatment wholeheartedly

Following the treatment wholeheartedly was necessary for it to be helpful. It was also essential to make the decision to challenge your fears and actually practice applying the skills. Some participants described that they did not put enough time and effort into the treatment, and experienced that fear and not being receptive to change prevented them from getting better.

*It is important to try to be actively involved and let the treatment come first, to do it at a hundred percent. (Participant 10)*

#### Skills for moving toward valued goals

Values were highlighted as being important for recovery, and skills as helpful for moving toward valued goals. Recurrent and continuous emphasis on values and valued goals helped patients remember and reflect on why they were doing certain things, such as gaining weight or challenging their fears.

*It’s like, what do I want to be like? Do I want to sit there when I’m 65, by myself, because no one wants to hang out with you any more, that’s not how you want your life to be. You want to be able to see that you’ve done fun stuff. (Participant 7)*

Various skills were pinpointed as helpful. Examples included working with chain analyses to learn how to see patterns, activating their parasympathetic nervous system in order to feel safe in social situations, participating without planning ahead, and using self-enquiry to learn more about oneself. Another example was enhancing intimacy in relationships through sharing personal experiences, e.g., by using a skill called “Match + 1” that helped the patient increase interpersonal warmth and closeness.

*I think it is called match, when you start small and go deeper and deeper in the relationship when you talk to someone … I did this when I met a friend, for like half a year, and in the end I was able talk about almost anything. (Participant 4)*

Patients described that not gaining adequate skills, for example for reducing anxiety, not following up on homework assignments, or not generalizing enough to the home environment might have hindered progress. Some felt that the material in skills class did not always fit their needs.

### The benefits of sharing and connecting with others

#### A trusting and genuine therapeutic relationship

A trusting, honest and open therapeutic relationship was important in the therapy. In particular, the open and genuine atmosphere was highlighted by the participants as being essential for sharing personal experiences and connecting with the therapist.

*It was nice that we had a genuine tone, If I did something my therapist thought was strange, she told me. And if I thought she did anything that was strange, I told her … that was helpful. (Participant 3)*

Patients also stated that they liked their therapist and felt that the therapist cared for and understood them. Several patients described the therapists as relaxed. It was seen as important that the therapist believed it was possible to get better; hope could fade if the therapist no longer communicated that change was possible. One description mentioned feelings of being reserved in therapy because of being scared of not being good enough or not being liked by the therapist.

*I don’t think it had anything to do with the therapist, I have trust issues and am afraid of not being liked … When I finally let my guard down and dared to show what I felt, then it was easier to feel trust. (Participant 1)*

Not being open to the therapist and not feeling like the therapist believed in change, could contribute to treatment drop-out.

#### Sharing and connecting with others in the group

The skills group provided an opportunity to practice sharing personal information and opinions with others. In particular, patients described sharing as important, saying it helped them recognize themselves in others and gain new perspectives and social connectedness. The experience of opening up and showing weakness – and still being accepted in the group – was seen as helpful.

*It was hard at first because you talked about sensitive stuff, but it was really rewarding hearing the others and recognizing myself in them … It was nice, you could help each other out, many of the things they said to me when I told them about things, I still remember and carry with me. (Participant 2)*

Patients welcomed that the instructors encouraged openness, and felt that the more they got to know the group, the better things got. If a new member joined, the group became more closed again for a while. Though most patients appreciated that the group was focused on life outside the ED, others wished for more opportunities to talk about the ED. One description mentioned not feeling safe in the group, contributing to treatment drop-out.

### Growing trust

#### Initial skepticism

There was an initial skepticism regarding various aspects of the therapy, e.g., sharing experiences and thoughts with the therapists and peers in the skills training. However, trust grew as the therapy progressed thanks to the perceived open and non-judgmental approach. Some questioned the contents of the treatment in the beginning, but this changed as the therapy progressed.

*In the beginning I felt like “these questions are really uncomfortable, I don’t want to do this”, that’s why I was skeptical in the beginning … I wanted solutions right away, like “This is what you should do” and “if I do that everything will be fine”. I know it doesn’t really work like that, but that’s what I wanted … But looking back it was really helpful. (Participant 5)*

Initial skepticism was partly based on the participants’ feelings of agony that came with exposure to something stressful. However, aversive feelings and physiological reactions also helped patients realize that something needed to be challenged. Openness in the group and the therapeutic relationship, as well as skills, helped them overcome such skepticism.

*In the beginning I was really suspicious and did not feel that I trusted my therapist … But the better I got, the more proof I got that what she did was good. And also … the more you open up to someone, a bond grows, whether you want it to or not. (Participant 3)*

#### Change takes time

Patients who completed treatment mentioned that change was not immediate, but growing as therapy progressed. It was important to highlight that things might get harder first and that the benefits could come later. Initially, confronting anxiety and gaining weight was difficult and it was important to highlight that a change in well-being would not come just because the weight began stabilizing.

*I did a lot of comparing: how fat I was, and if I had breakfast and felt full, that was the only thing I could think of. But then it gradually changed, and like in November I noticed “It’s been several days and I have been sitting on my bus and didn’t think about it, I’ve been thinking about other stuff” — but this was after several months of struggles. (Participant 2)*

### Moving toward valued goals – but some remain

#### Getting to know myself in a kind, but sometimes painful, way

Getting to know oneself in a kind way, even though it was sometimes painful, was perceived as a helpful process. The treatment gave the participants new perspectives, self-knowledge and self-awareness through self-enquiry. Reflecting on oneself was perceived as difficult: seeing who you really were, not just what you presented to others. Patients also described the experience of learning to embrace the pain and reflect, rather than escape. Many descriptions mentioned that participants liked themselves more and were kinder toward themselves after the treatment.

*Self-enquiry was one of the most positive things for me, to try and find the core, like “why does this make me sad or upset?” “What is this about?” Previously I kind of moved on and just dealt with things … but to take the time and find the essence is kind of a starting point to actually change, because you find out what really needs to change. (Participant 11)*

Receiving feedback on their social signaling was described as helpful to improve self-awareness and change overcontrolled behaviors.

*It’s like with the body language, like “yeah I have a quite closed body language … oh, so of course people don’t find it enjoyable to talk to me” … now I know that about myself and how to handle it. (Participant 3)*

#### A journey from rigidity to more flexibility, openness, and connectedness

Patients reported that they had gained improved flexibility and freedom, acting more from personal values than from a desire to be perfect and avoid distress. Some felt that the perfectionistic tendencies remained, but they became more able to make mistakes and did not act based on their fears to the same extent. Examples of participating without planning ahead and letting others do things in their own way were described. Changing from avoiding things to showing vulnerability, being more open and being able to show their real feelings, helped improve and deepen relationships.

*Even if it is hard to show it or talk about it, I really try to do it anyway … with some friends. Before, I tried to control other people’s picture of me a lot, I do that less now, I really try to show more of who I am … because I noticed that it has brought me stronger and better relationships. (Participant 4)*

#### Changes in the eating disorder

Patients described a normalization of their weight and less ED symptoms, improved flexibility and new perspectives on their body. For example, they described that their body was healthy, that they learned to listen to their body, and that their body was better suited for life in accordance with their personal values. Patients also described that they saw the connection between the ED and their overcontrol and relational difficulties, and that the eating had changed due to changes in the overcontrol behaviors.

*I’ve been able to let go of these thoughts about having a certain body and a certain weight, there are other things in life that are more important. There are days where I want to go with the eating disorder, but then I remind myself … “there are friends and school and things that I enjoy doing” … yeah, it’s really nice to have a life. (Participant 6)*

*There is nothing I can’t eat anymore, that is a huge difference … and when you don’t have as many “don’ts” you can be more social … and eat lunch with a friend, and it’s not so strict where and when. (Participant 8)*

#### … but some remain

Descriptions related to an absence of improvement mentioned that the ED changed character but had not improved, that it was too difficult to make changes, or an unwillingness to continue weight restoration, resulting in treatment drop-out. One description mentioned an intention of coming back to treatment after additional care. Patients sometimes described that they were healthier than before, but that some problems, such as weighing and measuring food at times or having issues with their body, remained. It was also noted that it was challenging to try new things, to start sharing experiences in social situations, and that it was scary and difficult to get closer to others.

*… there are some left, and I think it will take some time before that goes away too. I can get a lot of feelings of being fat at times … I guess that is what’s still left, and the fact that I need an approximate plan for my meal routines. (Participant 5)*

### Doing well in treatment

#### Not wanting to be a bother

The idea of potentially bothering the therapist was described as an obstacle to seeking contact and asking for help, especially in between sessions and through text messaging. Participants experienced that their difficulties with wanting to perform well or that they felt they were not doing well enough or were not liked by the therapist might have hindered them from seeking as much contact as they would have wanted.

*It felt difficult to contact her in between sessions, I felt like I was bothering her … I called a few times when I panicked and really wanted to deal with something. But this way of working between sessions was totally new to me … it could have been clearer, like “It’s not that I’m being nice to you, this is a part of my job, I want you to call”. (Participant 4)*

#### Being the best of patients

Wanting to be a good patient and do well in treatment was common, especially in comparison with others in the skills class. These ideas could be either helpful (e.g., pushing yourself in doing the homework or sharing in skills class) or unhelpful (e.g., not feeling like you were doing enough, or wanting to do too much, making the treatment stressful which might have contributed to treatment drop-out).

*It was good that there were other people in the group … it felt a bit like you’re not only disappointing yourself if you’re not working on a homework assignment, but the others in the group as well. (Participant 3)*

It was also considered helpful that it was seen as acceptable not to do everything perfectly in treatment. Social comparison in that area could be beneficial at times, since it was focused on adaptive skills, not on food or the ED.

*If someone hadn’t done something as well as the others in skills class, it was okay. I think that was a relief, since there wasn’t so much pressure on doing things perfectly. It’s like, what is a perfect treatment, should you be the perfect RO patient, or what?”. (Participant 5)*

## Discussion

The aim of this study was to evaluate patient experiences of RO DBT in outpatient treatment for AN.

In general, RO DBT was described as a flexible and complex treatment with a holistic view of the patients’ difficulties. More specifically, the combination of different treatment components, as well as the broader focus beyond ED symptoms, were highlighted as helpful. Indeed, treatments of AN have often been described by patients as having an excessive focus on food and weight, lacking a broad focus beyond the ED [7–9]. At the same time, it is known that re-nourishment and weight gain are essential to recover from AN [1, 3]. The findings in this study, with patients expressing appreciation for the fact that both their ED symptoms and their rigid, emotional, and relational difficulties were addressed, might indicate that RO DBT is a treatment that both is in line with the clinical guidelines and appeal to the patients.

Nevertheless, like in other outpatient treatments for AN [26–28], a significant number of patients (*n* = 5, 38%) dropped out from RO DBT. Three of those who dropped out participated in the interviews. There were no descriptions of the treatment per se as a bad fit. However, the interviews suggest that social comparison and not feeling safe in skills class might increase the risk for drop-out. Drop-out was also described as a consequence of difficulties in understanding the demanding material and in keeping a focus on overcontrol while being preoccupied with food and weight. These findings may correspond to research suggesting that a brain in starvation is less plastic and receptive to psychological treatments [29, 30]. However, day treatment programs [20] and inpatient treatment [19] with RO DBT for more severe AN have shown positive results. Further, it has been suggested that weight restoration and medical issues should be supported with appropriate interventions (e.g., medical management) while providing the RO DBT treatment [31, 32]. Based on the results in this study and in the single-case study of treatment effect, as well as previous research, it is reasonable to assume that RO DBT as outpatient treatment only (e.g., without additional meal support) might be sufficient for mild to moderate levels of AN and overcontrol. Patients with severe or enduring AN might be highly affected by starvation or other ED symptoms, making it difficult to fully engage in the RO DBT if delivered as the sole intervention. These patients are likely in need of additional support, e.g., by providing initial inpatient treatment or day program before or along the RO DBT treatment. A more flexible approach could have the potential to both reduce drop-out and improve clinical utility. Whether these adaptations affect drop-out and outcome has, however, not been evaluated. Moreover, the field is in need of additional evaluation. In particular, larger studies evaluating the efficacy of RO DBT for AN, in addition to contrasting it to other treatments, are warranted.

Another important aspect that emerged across several themes was the open and sharing atmosphere in both skills class and individual therapy. Most patients described the therapeutic relationship as good and helpful, highlighting that the therapists were genuine, open, relaxed, and able to understand them and that this helped them open up to their therapist and engage in treatment. This is in line with previous research, underlining the importance of an authentic and strong therapeutic relationship [7, 33] and its association with positive treatment outcome [34]. In RO DBT, enhanced openness and social signaling are also hypothesized to be important mechanisms for change toward a healthier life [16]. The patients’ perceptions of the value of sharing personal experiences in this study supported these ideas.

While some patients reported no improvement or merely a shift in how their ED difficulties appeared, several areas of improvement – such as a healthier weight, less ED symptoms, and stronger relationships – were described in most interviews. This also corresponds to the result from the single-case study of treatment effects [35], in which all eight completers, all of whom participated in this qualitative evaluation, displayed both healthy weight and ED symptoms within one SD of the community mean (i.e., no or minor residual ED symptoms) after treatment. Patients described increased self-awareness, more openness in their personal relationships, and increased flexibility. Interestingly, the effect on outcomes related to overcontrol was small in the single-case study of treatment effect. By reviewing qualitative data in addition to quantitative results, a more comprehensive picture of patient experiences of changes or lack of changes might be gained. For example, it is possible that the small effects in overcontrol in the quantitative evaluation were a result of unsatisfactory quantitative self-rating measurements. Moreover, it is likely easier to measure distinct ED symptoms such as restrictive or rigid eating, where validated instruments exist, than factors related to psychological well-being in general, and overcontrol in particular – such as flexibility and usage of social signaling. Findings in the present study underline the importance of developing quantitative instruments that can assess behavioral changes in relation to social signaling and an overcontrolled personality style.

### Strengths and limitations

Demographic and clinical data do not allow for any conclusions regarding severely underweight or male participants. Limitations also include the relatively low number of participants, especially patients dropping out from treatment. Thus, even though saturation was reached overall, it is likely that data on reasons for treatment drop-out were incomplete. Also, one of the therapists participated in the qualitative analyses, which might have affected how data were interpreted and analyzed. To address this issue, an experienced researcher without any involvement in the study was invited to perform the analyses. The diverse research group is also a strength, providing different backgrounds and perspectives.

## Conclusion and future research

This study is the first to provide a qualitative evaluation of outpatient RO DBT for AN, and one of few to evaluate the experiences of treatment for outpatient AN in general. The results indicated that patients appreciated the comprehensive treatment and the holistic view of their difficulties. In particular, patients described sharing as important for progress in all aspects of therapy: individual therapy, skills class, and in relationships outside therapy. Thus, patient experience suggest that a treatment with a main focus on overcontrol in AN is promising for treating the disorder. Future research should continue evaluating reasons for treatment drop-out, and identify valid quantitative measurements that can be used to assess behavioral changes in overcontrol. Research should also include evaluation of potential mechanisms of change – e.g., change in social signaling behaviors.

## Supplementary Information

**Additional file 1.**

## Data Availability

Data will not be made publicly available due to confidentiality, but can be made available upon reasonable request to the corresponding author.

## Abbreviations

AN: Anorexia nervosa
BMI: Body mass index
DSM: Diagnostic and statistical manual of mental disorders
ED: Eating disorder
RO DBT: Radically open dialectical behavior therapy
SD: Standard deviation
