# Supplementary material for: Sharing and connecting with others – patient experiences of radically open dialectical behavior therapy for anorexia nervosa and overcontrol: a qualitative study
Source: J Eat Disord. 2021 Mar 4;9:29. doi: 10.1186/s40337-021-00382-z (PMC7931519; doi:10.1186/s40337-021-00382-z)
Supplement: Supplementary file 1 — Additional file 1. [file 40337_2021_382_MOESM1_ESM.docx]

Appendix

| **Themes** | **Subthemes** |
| --- | --- |
| **A comprehensive treatment** | A flexible and complex approach  Following, or not following, the treatment wholeheartedly  Skills for moving toward valued goals |
| **The benefits of sharing and connecting with others** | A trusting and genuine therapeutic relationship  Sharing and connecting with others in the group |
| **Growing trust** | Initial skepticism  Change takes time |
| **Moving toward valued goals – but some remain** | Getting to know myself in a kind, but sometimes painful, way  A journey from rigidity to more flexibility, openness, and connectedness  Changes in the eating disorder  … But some remain |
| **Doing well in treatment** | Not wanting to be a bother  Being the best of patients |
